# Short tandem repeat sequences in the *Mycoplasma genitalium *genome and their use in a multilocus genotyping system

**DOI:** 10.1186/1471-2180-8-130

**Published:** 2008-07-29

**Authors:** Liang Ma, Stephanie Taylor, Jørgen S Jensen, Leann Myers, Rebecca Lillis, David H Martin

**Affiliations:** 1 Section of Infectious Diseases, Department of Medicine, Louisiana State University Health Sciences Center, New Orleans, LA 70112, USA; 2 Mycoplasma Laboratory, Statens Serum Institut, DK-2300 Copenhagen S, Denmark; 3 Department of Biostatistics, Tulane University, New Orleans, LA 70112, USA

## Abstract

**Background:**

Several methods have been reported for strain typing of *Mycoplasma genitalium*. The value of these methods has never been comparatively assessed. The aims of this study were: 1) to identify new potential genetic markers based on an analysis of short tandem repeat (STR) sequences in the published *M. genitalium *genome sequence; 2) to apply previously and newly identified markers to a panel of clinical strains in order to determine the optimal combination for an efficient multi-locus genotyping system; 3) to further confirm sexual transmission of *M. genitalium *using the newly developed system.

**Results:**

We performed a comprehensive analysis of STRs in the genome of the *M. genitalium *type strain G37 and identified 18 loci containing STRs. In addition to one previously studied locus, MG309, we chose two others, MG307 and MG338, for further study. Based on an analysis of 74 unrelated patient specimens from New Orleans and Scandinavia, the discriminatory indices (DIs) for these three markers were 0.9153, 0.7381 and 0.8730, respectively. Two other previously described markers, including single nucleotide polymorphisms (SNPs) in the rRNA genes (rRNA-SNPs) and SNPs in the MG191 gene (MG191-SNPs) were found to have DIs of 0.5820 and 0.9392, respectively. A combination of MG309-STRs and MG191-SNPs yielded almost perfect discrimination (DI = 0.9894). An additional finding was that the rRNA-SNPs distribution pattern differed significantly between Scandinavia and New Orleans. Finally we applied multi-locus typing to further confirm sexual transmission using specimens from 74 unrelated patients and 31 concurrently infected couples. Analysis of multi-locus genotype profiles using the five variable loci described above revealed 27 of the couples had concordant genotype profiles compared to only four examples of concordance among the 74 unrelated randomly selected patients.

**Conclusion:**

We propose that a combination of the MG309-STRs and MG191-SNPs is efficient for general epidemiological studies and addition of MG307-STRs and MG338-STRs is potentially useful for sexual network studies of *M. genitalium *infection. The multi-locus typing analysis of 74 unrelated *M. genitalium*-infected individuals and 31 infected couples adds to the evidence that *M. genitalium *is sexually transmitted.

## Background

*M. genitalium *has been recognized as an important cause of nongonococcal urethritis (NGU) in men and is likely to be associated with genital tract inflammatory diseases in women, such as cervicitis [1,2], endometritis [3], pelvic inflammatory disease [4], and tubal factor infertility [5]. The epidemiologic data suggests that *M. genitalium *is a sexually transmitted pathogen [2,6-9]. A recent molecular study supports these findings [10].

Study of *M. genitalium *presents several unique challenges. It remains extremely difficult to isolate the organism from clinical specimens, and thus, identification of infected individuals is dependent on the use of polymerase chain reaction (PCR) tests. *M. genitalium *samples derived directly from patients are usually contaminated with human cells and other microbes, and may contain PCR inhibitors [11,12]. These difficulties have impeded progress in understanding the epidemiology and pathogenic role of *M. genitalium*. During the last few years, several molecular methods have been reported to be useful for strain typing, including: short tandem repeat (STR) analysis of putative lipoprotein (PLP) genes [13], single nucleotide polymorphisms (SNPs) in the rRNA genes [13], restriction fragment length polymorphisms (RFLP) of the MG192 (*mgpC*) gene [14], and SNPs in the MG191 (*mgpB*) conserved gene [10,12]. The value of these typing techniques has never been comparatively assessed. The aims of this study were: 1) to identify new potential genetic markers based on an analysis of STRs in the published *M. genitalium *genome sequence; 2) to compare these newly identified markers and those previously described for their utility as components of an efficient multi-locus genotyping system; 3) to provide further evidence for sexual transmission of *M. genitalium*.

## Results

### Bioinformatics analysis of tandem repeats in the *M. genitalium *G37 genome

Although the presence of tandem repeats in the *M. genitalium *genome has been noted in previous studies [13,15,16], a comprehensive list of tandem repeats with accurate information on the location and repeat unit sequence is not available. We performed a computerized search of the *M. genitalium *G37 genome [17] and identified 18 loci containing tandem repeat units ranging from 1 to 5 bases in length (Table 1). None of these loci had repeat unit lengths longer than 5 bases (therefore they are all STRs). The repeat copy number for the 18 STR loci varied from 4 to 26. The majority of these STRs (15/18) consisted of a trinucleotide repeat unit. For the remaining three loci repeat units consisted of a mononucleotide, tetranucleotide or pentanucleotide in one each. Nine STRs were located in coding regions while all others fell in non-coding regions. Six of the non-coding region STRs were located in MgPa-related repetitive elements (MgPars) [17,18], including MgPar 1, MgPar 2, MgPar 8 and MgPar 9 [19]. A trinucleotide AGT repeat motif was present in 6 loci, with one in the coding region of MG307, two in the coding region of the adhesin MgPa operon (MG191 or *mgp*B and MG192 or *mgp*C genes), and three in MgPar regions (including MgPar 2, MgPar 8 and MgPar 9). While the AGT repeat unit has been previously annotated as TAG repeat by other investigators based on analysis of the G37 genome sequence only [15,16,19], our analyses of multiple *M. genitalium *strains in the present study and in an earlier study [20] have demonstrated that the correct annotation is AGT. The repeat unit TCT identified in MgPars 1, 8 and 9 in this study has been annotated differently in previous reports [15,16], either as CTT or AAG (complementary to CTT). We have found evidence that the TCT repeat region in these three MgPars can recombine with the MG192 gene in which they encode a stretch of serines [20]. Thus we believe that the correct repeat unit sequence should be TCT. The STR sequence in MG309 was shown to be a mixture of AGT and AAT trinucleotides in our previous study [13] rather than a hexanucleotide repeat (TATTAC in antisense direction) as previously reported [16]. All STR regions except for those in MG192 and MgPars were present in a single copy in the genome.

**Table 1:** STRs in the *M. genitalium *G37 genome (GenBank no. NC_000908).

Repeat position on genome	Repeat unit	Copy no.	Affected gene or locus^a^
36790–36808	A	19	MG031–MG032
86053–86067	TCT	5	MgPar 1
169476–169523	AGT	16	MgPar 2
198766–198777	TTG	4	MG168 (*rps*E)
203554–203569	CAAC	4	MG177 (*rpo*A)
212934–212953	AAACA/AAACC	4	MG185 (lipoprotein)
224535–224555	AGT	7	MG191 (*mgp*B)
227131–227163	AGT	11	MG192 (*mgp*C)
273587–273598	TAG	4	MG226 – MG227
313502–313513	TAG	4	MG260–MGt17
349736–349750	TCT	5	MgPar 8
351453–351482	AGT	10	MgPar 8
378382–378396	AGT	5	MG307 (lipoprotein)
384453–384488	AGT/AAT	12	MG309 (lipoprotein)
425824–425856	ACA	11	MG338 (lipoprotein)
429309–429335	AGT	9	MgPar 9
429967–430047	TCT/ACT	26	MgPar 9
542533–542544	TTG	4	MG437

^a^Given as the locus tag annotated in GenBank or in reference [19].

### Use of short tandem repeats in a multilocus genotyping system

Previous studies have found that the STRs in MG191, MG192 and MgPars undergo extensive intrastrain variation due to DNA recombination and/or other mechanisms [[20,21], Ma *et al*., unpublished data], indicating that these STRs are unsuitable for strain typing. Because our earlier study showed that the STR in the MG309 PLP gene was highly variable among clinical strains [13], we targeted the other three PLP loci (MG185, MG307 and MG338) for further investigation. The remaining 6 STR loci were not evaluated due to limited volume of the DNA extract available from the specimens. Thirty-one unrelated New Orleans specimens were studied initially which revealed significant variation in MG307 and MG338 but none in MG185. Therefore the latter locus was excluded from further study.

**Figure 1:**
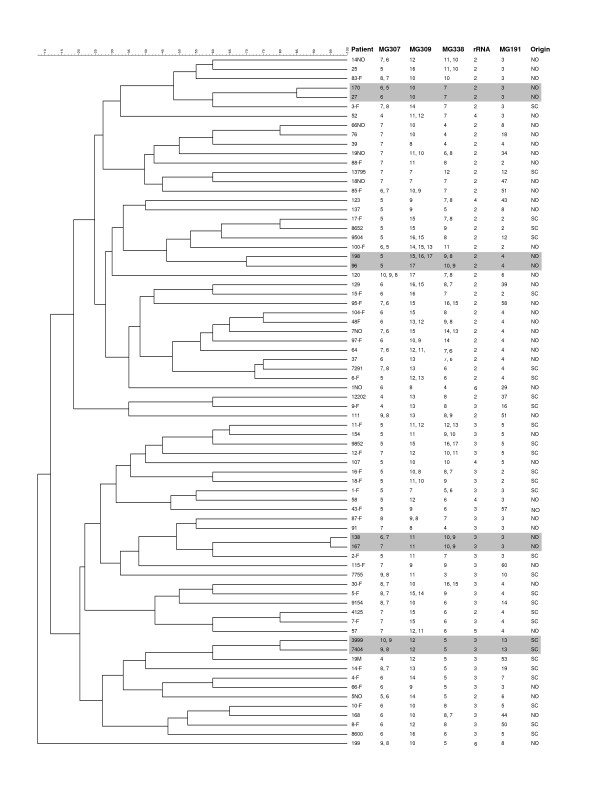
**Clustering dendrogram of unrelated *M. genitalium *clinical strains based on genotype profiles at five genomic loci**. The dendrogram was constructed using categorical coefficient and UPMGA clustering and included 30 strains from Scandinavian patients (SC) and 44 from New Orleans patients (NO). The genotypes at three PLP STR loci were determined by direct sequencing alone. For specimens containing mixed STR sequences, the genotypes are listed in the order from predominant to minor type. Only 4 sets of two patients had matching genotype profiles at all 5 loci (highlighted in grey shading). Note that three sets of two patients (nos. 58 and 14NO, 104F and 7NO, and 66NO and 199) and one set of three patients (nos. 27, 170 and 83F), which showed identical MG309-STR and MG191-SNP genotypes, could be distinguished by MG307-STRs, MG338-STRs or rRNA-SNPs.

**Figure 2:**
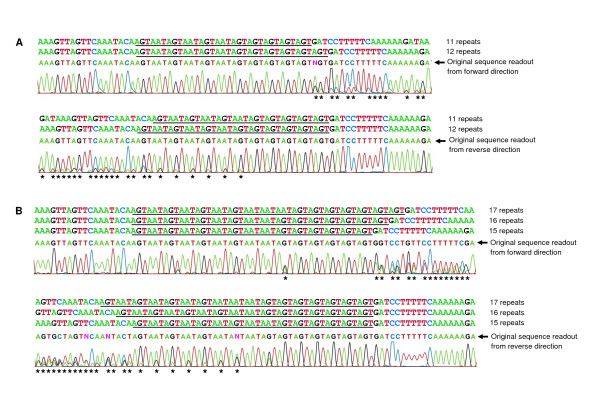
**Representative sequence chromatograms illustrating how mixed STR sequences can be detected by direct sequencing**. (**A**) Partial sequence chromatograms of MG309-STRs in specimen no. 57 which contained a mixture of 12 and 11 repeats. (**B**) Partial sequence chromatograms of MG309-STRs in specimen no. 198.2 which contained a mixture of 15, 16 and 17 repeats. The PCR product from each specimen was directly sequenced in two separate sequencing reactions from forward and reverse directions, respectively. The AGT/AAT repeat units are underlined. In direct sequencing of the PCR products, the minority population (11 repeats in panel A and 16 and 17 repeats in panel B) is three bases out of alignment with the majority population (12 repeats in panel A and 15 repeats in panel B), resulting in a mixture of two or three chromatogram peaks as indicated by asterisks. The sequences flanking the repeat region can be determined accurately by sequencing from two directions. The estimated individual sequences shown above the original sequence readouts were confirmed by sequencing of plasmid clones for both specimens (no. 57 in additional file 2 and no. 198.2 in additional file 2 and Figure 3B).

The variability of MG307, MG309, and MG338 STRs was determined in a total of 44 unrelated New Orleans patients and 30 unrelated Scandinavian patients (Figure 1). Among the 74 patient samples studied, the number of repeats varied from 4 to 10 for the MG307-STRs, 7 to 17 for the MG309-STRs, and 3 to 17 for the MG338-STRs. Twenty-eight (37.8%) patients contained a single allele at all three STR loci. Sequence analysis revealed a mixture of two or more STR alleles in the three PLP loci as follows: MG307 – 30%, MG309 – 23%, and MG338 – 32%. A total of 46 (62%) of the specimens had mixed STR sequences in at least one of the STR-containing loci. In all mixed sequences, the number of repeats varied by only one or two repeat units. The prevalence of mixed sequences in both MG307-STRs and MG338-STRs significantly increased as the repeat copy number became larger (Spearman's correlation coefficient = 0.87 to 0.95, *P *< 0.0001) while this correlation was not significant for the MG309-STRs (Spearman's correlation coefficient = 0.26, *P *> 0.45. See additional file 1). The STR numbers in specimens with mixtures of sequences were determined readily by direct sequencing of the PCR products in the forward and reverse directions, in which individual sequences were read by direct observation of the sequence chromatograms (Figures 2A and 2B).

To verify the presence of mixed sequences as determined by direct sequencing, we performed subcloning of PCR products from 13 selected specimens containing mixed sequences, followed by sequencing of 4–19 individual plasmid clones per specimen. The presence of a mixture of 2 or more sequences as determined by direct sequencing was verified in all 13 specimens (See additional file 2). Plasmid cloning revealed the presence of a third and sometimes a fourth sequence in addition to those identified by direct sequencing in a few cases. In one sample that showed no evidence of a mixed STR population by direct sequencing, the 12 plasmid clones that were sequenced also showed no evidence of sequence mixtures (specimen no. 123.1; see additional file 2). In addition, for 20 selected specimens showing mixed sequences we repeated PCR using DNA polymerases with proof-reading activity and obtained sequences by direct sequencing that were the same as those provided by *Taq *DNA polymerase. This provided evidence that mixed PLP STR sequences cannot be attributed to PCR artefacts.

**Figure 3:**
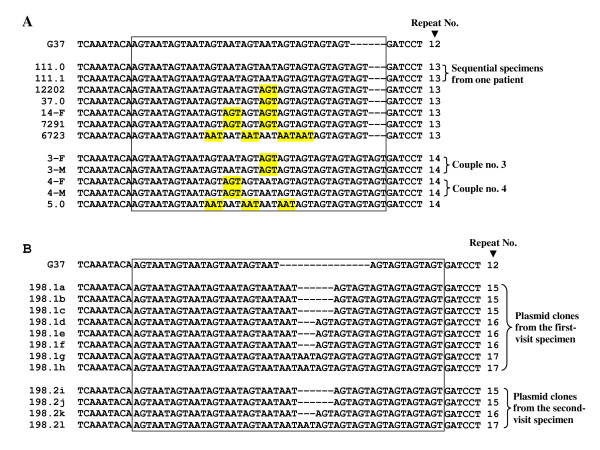
**Variation in repeat numbers and distribution patterns of the MG309 AGT/AAT repeat sequence**. (**A**) MG309 AGT/AAT repeat sequences in selected patient specimens obtained from unrelated patients, sequential specimens from the same patients and specimens from concurrently infected couples. (**B**) MG309 AGT/AAT repeat sequences among selected plasmid clones from two sequential specimens from one patient (no. 198). The published sequence from the *M. genitalium *G37 genome (GenBank accession number NC_000908) was chosen as the reference sequence (G37). The boxed regions in each panel contain the MG309 AGT/AAT repeat units. Hyphens indicate gaps introduced to optimize alignment. Numbers on the left of each sequence refer to the specimen codes (panel A) or plasmid clones (panel B). Repeat units that differ from those of G37 are highlighted in yellow.

Because MG309-STRs contain two different repeat units, AGT and AAT, we examined the distribution patterns of these units among different specimens. Among 57 unrelated patient specimens (including 33 New Orleans patients and 24 Scandinavian patients), which showed no evidence of sequence mixtures, the number of MG309 repeats varied from 7 to 17. Two to four different AGT/AAT distribution patterns were observed in specimens having the same number of STRs with the exception of the five specimens with 9 repeats, which were all identical. When both the repeat number and distribution pattern variations were taken into account, there were 29 unique MG309 sequence types among these 57 patients. Figure 3A shows an example of the variation in the distribution of the AGT and AAT repeat units among patient specimens containing 13 and 14 repeats. The distribution patterns of these repeat units were stable in sequential specimens from the same patients (Figure 3A) and in plasmid clones that had an identical number of repeats and were obtained from the same patients (Figure 3B).

### rRNA single nucleotide polymorphism (SNP) typing

**Figure 4:**
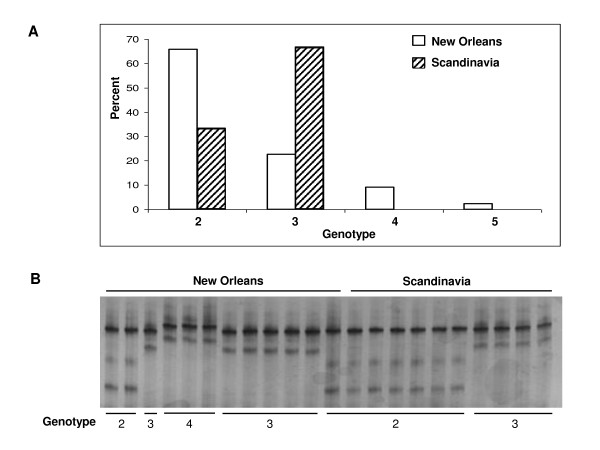
**Molecular typing of *M. genitalium *based on rRNA-SNPs**. **(A) **SSCP analysis of a 246-bp DNA fragment covering the three polymorphic sites for types 2, 3 and 4 of the rRNA operon [13]. The gel was sliver stained. **(B) **Distribution of *M. genitalium *rRNA-SNP genotypes between New Orleans (n = 44) and Scandinavia (n = 30) patients. The DNA sequences for types 2 to 5 are available from GenBank with accession numbers AY374418 to AY374421. By the generalized Fisher's exact test, *p *= 0.0004 for the overall distribution. By traditional Fisher's exact test, *p *= 0.0089 for type 2 and *p *= 0.0002 for type 3.

Overall rRNA-SNPs types 2 and 3 are most common (52.7% and 40.5%, respectively). Types 4 (5.4%) and 5 (1.4%) are relatively uncommon. As in our previous study, Type 1 is not present. This type is uniformly found in the currently available ATCC strains but apparently nowhere else. Stratifying the data geographically revealed a significant difference between rRNA-SNP types in New Orleans and Scandinavia (Figure 4A). In New Orleans Type 2 is most common (65.9%) while in Scandinavia 3 is the predominant type (66.7%) (*P *< 0.0004 by Fisher's exact test). Thus far types 4 and 5 are seen only in New Orleans. Of interest is that none of the other typing loci studied here differ in prevalence between the two locations.

To increase efficiency of rRNA-SNP locus typing we developed a simple and rapid method based on single-strand conformational polymorphism (SSCP) analysis to detect the rRNA-SNPs. As shown in Figure 4B, Types 2, 3 and 4 exhibited different SSCP patterns. Type 2 showed three bands which were well separated. In contrast, types 3 and 4 showed two bands with different mobility. Subsequently, we applied the SSCP assay blindly to all additional specimens used in this study. The typing results using the SSCP assay were completely consistent with the sequencing data for these three genotypes.

### MG191-single nucleotide polymorphism typing

This typing method is based on sequence analysis of the polymorphic sites in an approximately 280-bp fragment of the MG191 conserved AB region [10]. Previous study of 267 specimens by this method has identified 56 unique genotypes, designated type 1 to 56 [10]. In this study, we observed 19 and 15 different sequence types in 44 unrelated New Orleans patients and 30 unrelated Scandinavian patients, respectively (Figure 1). When the results for both patient populations were combined, there were a total of 27 unique sequence types. Twenty-three of these were identical to those reported previously by Jensen *et al*. [10]. Among 11 previously unstudied specimens from New Orleans patient four new types (types 57 to 60) were identified, which have been deposited into GenBank with accession numbers EU131379 to EU131382.

### Stability of various genetic markers

Two sequential specimens were obtained from each of 10 New Orleans patients at an interval of 10 to 36 days (Table 2). None of the 10 patients showed a change in MG309-STRs, rRNA-SNPs or MG191-SNPs. Two patients had minor changes in the STR number at MG307 and/or MG338. These changes involved either contraction or expansion of only one repeat unit.

**Table 2:** Stability of *M. genitalium *genotypes in sequential samples from 10 patients.

Patient	Sample	Interval (day)	No. of repeats^a^			rRNA-SNP	MG191-SNP
					
			MG307	MG309	MG338		
64	1	11	7, 6	12, 11, 10	7, 6	2	4
	2		7, 6	12, 11, 10	7, 6	2	4
91	1	34	7	8	4	3	3
	2		7	8	4	3	3
111	1	36	9, 8	13	8, 9	2	51
	2		9, 8	13	8, 9	2	51
123	1	21	5	9	7, 8	4	43
	2		5, 6	9	7	4	43
129	1	12	6	16, 15	8, 7	2	39
	2		6	16, 15	8	2	39
137	1	26	5	9	5	2	8
	2		5	9	5	2	8
168	1	15	6	10	8, 7	3	44
	2		6	10	8, 7	3	44
170	1	23	6, 5	10	7	2	3
	2		6, 5	10	7	2	3
198	1	19	5	15, 16, 17	9, 8	2	4
	2		5	15, 16, 17	9, 8	2	4
199	1	10	9, 8	10	5	6	8
	2		9, 8	10	5	6	8

^a ^Underlined numbers indicate repeats confirmed by sequencing of cloned PCR products (See additional file 2). For specimens containing mixed sequences, the genotypes are listed in the order from predominant to minor type.

### Discriminatory power of various genetic markers

The discriminatory index (DI) for each marker was calculated using the 28 specimens having a single allele at all five loci studied (See additional file 3). Of the five markers evaluated, the MG191-SNPs and the MG309-STRs loci had the highest DIs (0.9392 and 0.9153, respectively), followed by the MG338-STRs and MG307-STRs (0.8730 and 0.7381, respectively). The rRNA-SNPs had the lowest DI (0.5820). When the variation in both the repeat number and the distribution of the AGT and AAT repeat units at the MG309 locus was considered, the DI increased to 0.9471. We analyzed the DI for various combinations of two of the 5 markers and found that the combination of MG309-STRs and MG191-SNPs gave the highest (0.9894). However, some specimens containing identical MG309-STR and MG191-SNP genotypes (including three sets of two specimens and one set of three specimens), still could be distinguished by MG307-STRs, MG338-STRs or rRNA-SNPs (Figure 1).

### Analysis of sexual transmission by multi-locus typing

**Figure 5:**
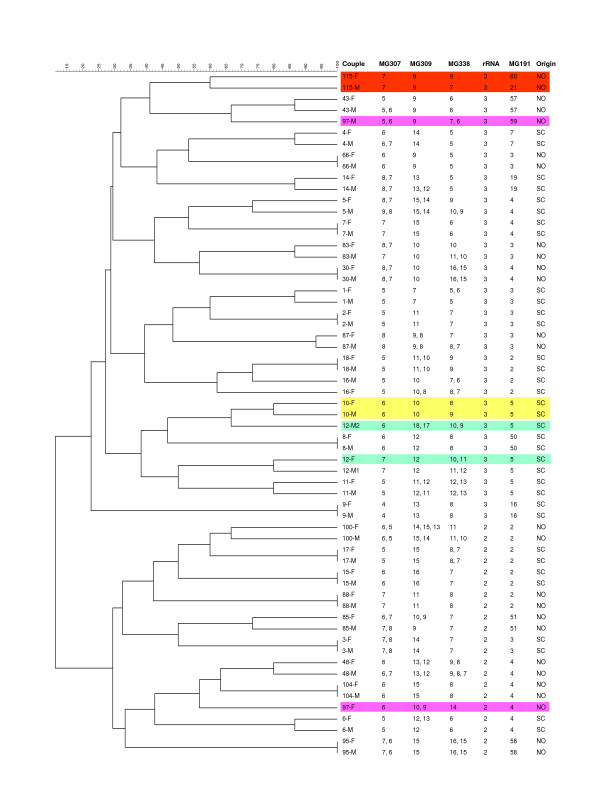
**Clustering dendrogram of *M. genitalium *genotype profiles in concurrently infected couples**. Eighteen couples were Scandinavia (SC) and 13 were from New Orleans (NO). The dendrogram was constructed using categorical coefficient and UPMGA clustering. Each couple was assigned the same number. F – female; M – male. One female (12-F) had two male partners 12-M1 and 12-M2. For specimens containing mixed sequences, the genotypes are listed in the order from predominant to minor type. The 4 couples showing mismatching profiles were highlighted in four different colours.

To study the sexual transmission of *M. genitalium *infection, we analyzed the genotype profiles at the five variable loci described above in specimens obtained from 31 concurrently infected couples (18 couples from Scandinavia and 13 from New Orleans) in comparison with the 74 unrelated patients. Figure 1 shows the genotype profile at 5 loci for 74 unrelated patients. For each patient, the genotypes at all 5 loci were used to define the multi-locus genotype profile. Patients were classified as having matching profiles if they had identical alleles at all loci or if they had identical alleles at rRNA-SNP and MG191-SNP loci and shared at least one common allele at each of the three variable STR loci when mixed STR alleles were present. Among these 74 patients, only four sets of two patients had matching profiles and all the remaining patients had mismatching profiles. Among the 31 couples studied (Figure 5), matching genotype profiles were identified within 27 couples. Among the four couples without complete matches, one (no. 10) had a mismatch of one repeat unit at a single PLP locus (MG338) while three (nos. 12, 97 and 115) had mismatches at two or three loci. For all couples showing matching genotypes at MG309 STRs, the distribution patterns of the AGT/AAT units were also identical.

## Discussion

In this study, we carried out a comprehensive analysis of the STRs in the genome of the *M. genitalium *type strain G37 and found two new genetic markers that are potentially useful for molecular typing of this organism. We evaluated the discriminatory power of the new STR markers (MG307 and MG338) and three previously described markers, including the MG309-STRs [13], rRNA-SNPs [13] and MG191-SNPs [10], using a collection of 74 unrelated patient specimens from both Scandinavia and New Orleans. We found that the MG191-SNPs and the MG309-STRs had high discriminatory power (see additional file 3) while MG338-STRs, MG307-STRs and rRNA-SNPs were less so though still useful. The discriminatory power of MG309-STRs was further improved by the variation in the distribution patterns of the two types of repeat units. A combination of MG309-STRs and MG191-SNPs yielded almost perfect discrimination (DI = 0.9894). In addition, we found that though the rRNA-SNPs marker had a very low DI, its geographical distribution differed strongly between Scandinavia and New Orleans. Finally, we applied multi-locus typing to study the sexual transmission in concurrently-infected couples from Scandinavia and New Orleans and further confirmed that *M. genitalium *is sexually transmitted [10].

Molecular typing has proven to be an important tool for understanding the epidemiology and pathogenesis of bacterial infections. During the past few years, multiple attempts have been made to develop strain typing methods for *M. genitalium*. Kokotovic *et al*., were the first to describe a typing method for *M. genitalium*, which is based on whole-genome fingerprinting involving selective amplification of restriction fragments [22]. This method requires purified DNA from cultured strains and is, therefore, not applicable to typing directly from clinical specimens. To overcome this limitation, several new methods have been reported, all of which rely on PCR amplification of a specific genomic locus followed by DNA sequencing or RFLP analysis. They include STR analysis of the PLP gene MG309 [13], SNPs of the rRNA operon [13] and the MG191 (*mgpB*) gene [10], and RFLP of the MG192 (*mgpC*) gene [14]. The target for the MG192-RFLP system developed by Musatovova *et al*. [14] involves a region that has multiple copies (termed MgPar repeats) dispersed throughout the *M. genitalium *genome. We and others have found that this region is undergoing rapid sequence shifts over time in vitro and in vivo due to homologous recombination with MgPar repeats [20,21], suggesting that the MG192-RFLP system [14] is not suitable for *M. genitalium *genotyping. The other three typing methods, including MG309-STRs, rRNA-SNPs [13] and MG191-SNPs [10], appear to have high stability and excellent discriminatory power. However, they have never been directly compared with respect to discriminatory power and utility in a multilocus genotyping system.

The present study is the first to compare the discriminatory power of different genotyping markers for *M. genitalium *based on data derived from the same collection of specimens. We found that MG309-STRs and MG191-SNPs had greater discriminatory power than other markers. The major advantage of STR typing is that it gives the results as numbers allowing for easy interpretation and exchange of data between different laboratories. A disadvantage of STRs typing is the presence of mixed repeat patterns in approximately 20–34% of clinical strains, which may complicate the interpretation of the results for laboratory personnel inexperienced in reading sequence chromatograms. Investigation of the possibility of separating the mixed sequences and quantifying the copy number by other approaches such as denaturing high-resolution gel electrophoresis [23] or fluorescent fragment sizing [24] would be worthwhile. Because SNPs and STRs differ in molecular mechanisms and rates of mutation, combined use of SNPs and STRs can complement each other, provide higher typing efficiency and may define more accurate genetic relationships [25]. Of the five markers evaluated in this study, MG191-SNPs showed the highest DI among SNP loci while MG309 STRs showed the highest DI among STR loci (see additional file 3). The discriminatory power of the MG309 STRs locus is further improved by the variation in the distribution patterns of the two types of repeat units (DI = 0.9471). A combination of MG309-STRs and MG191-SNPs achieved almost perfect discrimination (DI = 0.9894). We propose that a combination of MG309 STRs and MG191-SNPs would work efficiently for general epidemiological studies of *M. genitalium *infection. The addition of the other three loci results in a small increase in discriminatory power but requires increased effort and expense.

It is of interest that the distribution of the rRNA-SNPs differs strongly between Scandinavia and New Orleans (Figure 4A) whereas MG191-SNPs and PLP gene STRs did not (data not shown). Even with the limited number of specimens studied thus far, 60 genotypes of the MG191-SNPs have been described thus far [10]. In contrast, only 6 types of rRNA-SNPs have been found [13]. This is consistent with the known slow rate of rRNA mutation making this gene less useful in dynamic sexual network studies but more useful for tracking geographic distribution of *M. genitalium*. It would be of considerable interest to determine the rRNA-SNPs distribution in diverse populations from around the world. Moreover, it would be worth determining whether or not rRNA-SNPs associations correlate with environmental factors and/or bacterial virulence.

While the PLP STRs are stable over a period of up to 5 weeks (Table 2), high frequency of single repeat unit variants resulting in the appearance or disappearance of mixed STR alleles suggests the existence of clonal variants resulting from high mutation rates in these STR loci. These are generally believed to be caused by slipped strand mispairing during DNA replication [26]. The finding that mixed STR alleles are strongly associated with larger repeat copy numbers in MG307-STRs and MG338-STRs suggests that larger repeat copy numbers have higher mutation rates, consistent with studies of STR mutations in other bacteria [27,28]. In each of those specimens containing mixed STR alleles, the individual alleles differed by only one or two repeat units and there was no evidence of mixed alleles in MG191-SNPs or rRNA-SNPs, further supporting the contention that the presence of mixed STR alleles are not due to coinfection with two or more different strains. Therefore, when using the STR containing loci for epidemiological purposes, if any two samples are found to share one or more common STR alleles they should be considered to have the same genotype at that locus.

Sexual transmission of *M. genitalium *has been suggested by a number of epidemiological studies which have found either high infection concordance rates among sexual partnerships [6-8,29] or association with high risk sexual behaviour [2,30,31]. However, such epidemiological studies suggest but do not prove sexual transmission of the organism. Other methods are needed to unequivocally establish the sexual transmission of *M. genitalium *infection. We have previously addressed this issue by MG191-SNP analysis of 19 concurrently-infected couples [10]. For all 19 couples the sequence type found in specimens from the male partner was identical to that in the female partner. In one of these couples a new sequence type was documented to have been introduced by a female to her male partner. In the present study we investigated the genotype profiles at five genomic loci in specimens from 74 unrelated patients and 31 concurrently infected couples. Among the 74 unrelated patients, only four sets of two patients had matching genotype profiles. In contrast, among the 31 couples, matching genotype profiles were identified within 27 couples and only 4 couples showed mismatching profiles. Furthermore, each of the 27 matched couples shared a unique genotype profile. The degree of concordance (87.1%) in this study is similar to that (90.3%) reported for *Neisseria gonorrhoeae *infection from sexual contacts, which was determined by multi-antigen sequence typing [32]. These findings clearly demonstrate that unrelated patients are infected by different strains and that the concurrently infected couples are infected by the same strains thus proving sexual transmission of *M. genitalium*. It is noteworthy that, despite the presence of mixed alleles in at least one of the three variable STR loci in 18 couples, mismatching MG338 and MG309 STR copy numbers were observed between the members of only two couples (12 and 97), while all the remaining couples showed matched repeat copy numbers (at least one copy number identical). This suggests that the clonal variant populations are continuously transmitted between regular sexual partners through repeated exposure. The stability of these highly discriminatory genotypes suggests that STR analysis might be useful for sexual network studies of *M. genitalium *infection.

## Conclusion

We evaluated the performance of several genetic markers for typing of *M. genitalium *and found that the MG191-SNPs and the MG309-STRs had comparable high degrees of discriminatory power. A combination of MG309-STRs and MG191-SNPs achieved almost perfect discrimination. The ability to find concordant genotypes within concurrently infected couples and different genotypes among unrelated patients suggests that these typing methods have excellent epidemiological concordance. We propose that a combination of the MG309-STRs and MG191-SNPs is efficient for general epidemiological studies and addition of MG307-STRs and MG338-STRs is potentially useful for sexual network studies of *M. genitalium *infection.

## Methods

### Bioinformatics analysis of tandem repeats in the *M. genitalium *genome

In an attempt to increase the repertoire of previously established STR markers [13], we screened the complete genome of the *M. genitalium *type strain G37 [17] for tandem repeats using the Tandem Repeats Finder program [33]. The program generates an output file giving the repeat location, the repeat unit length, the copy number, and the nucleotide composition. We used the selection criteria recommended by the program as follows: repeat unit length, 1–100 bp; minimal copy number 11 for single base repeats, 5 for 2-bp repeats, 4 for 3- to 9-bp repeats, 3 for 10- to 100-bp repeats; percent matches, 95–100.

### *M. genitalium *specimens

Two sets of *M. genitalium *positive specimens were used in this study (Figure 1 and Figure 5). The first set was obtained between 1997 and 2002 from patients in Scandinavia, including 18 concurrently infected couples (one female had two male partners) and 30 unrelated patients (including the female from each couple), who had been described previously in a MG191 sequence-based typing study [10]. The second set was obtained between 2002 and 2005 from patients in New Orleans, Louisiana, USA, including 13 concurrently infected couples and 44 unrelated patients (including the female from each couple). Thirty-one of the 44 unrelated patients were described along with their rRNA-SNPs and MG309 STRs genotypes in our earlier study [13]. For the New Orleans patients, specimens were first voided urine from men and cervical swabs from women. The average time between recovery of specimens from the 31 couples was 7.5 days (ranging from 0 to 90 days), and 74.2% of the specimens were recovered within a week of each other. Genomic DNA was extracted directly from clinical specimens by using the Chelex 100 Resin (Bio-Rad, Hercules, CA, USA) or the High Pure PCR Template Preparation Kit (Roche Applied Science, Indianapolis, IN) as described elsewhere [12,31]. This study was approved by the relevant ethical committees.

### PLP-STR typing

PCR amplification and sequence analysis of the MG309-STRs in 31 of the 44 unrelated patients from New Orleans was reported in an earlier study [13]. In the present study we used the same primers and conditions described previously to amplify the MG309-STRs in the new specimens included in this study. To amplify the STRs in MG185, MG307 and MG338, primers were designed based on the sequences flanking the STRs of these three loci by using the Primer3 software [34] (Table 3). To obtain PCR products in sufficient quality and quantity for sequencing, we carried out a primary PCR for all samples and a nested PCR for those samples for which the initial PCR products were not visible or very faint on agarose gels. The primers used to amplify the MG185 STR region were 185F1 and 185R1 for the primary PCR and 185F1 and 185R2 for the nested PCR. The primers used to amplify the MG307 STR region were 307F6 and 307R3 for the primary PCR and 307F5 and 307R4 for the nested PCR. The primers used to amplify the MG338 STR region were 338F1 and 338R5 for the primary PCR and 338F4 and 338R4 for the nested PCR. Amplification was conducted on a PTC-200 programmable thermal controller (MJ Research, Inc., Watertown, MA, USA). PCR conditions were same as previously described for amplifying the MG309-STRs [13], except that the annealing time was reduced to 1 min for all reactions.

**Table 3:** Primers designed in this study.

Primer name	Sequence (5'→3')	Target
185F1	CAACTGCACGCTTTCCCTAT	MG185
185R1	CCTCTCTTTCATTTGCATGG	MG185
185R2	GCGTTGGGGTGCTTTTAGAAC	MG185
307F6	TGACCATTATCGCTTCATTT	MG307
307R3	AGTTGTTGGGTGCCTGTAT	MG307
307F5	GTAACAACACCAATAATCCTCAG	MG307
307R4	TTGGTTGGTTTGGGTACTTC	MG307
338F1	GGTGGTCAAACAAACTCTGC	MG338
338R5	CCTCCATCAATATGAAATGC	MG338
338F4	AAGTATAGTGCAAGCAAAGT	MG338
338R4	CTTGTTACTACTTGTGCTTGAGATG	MG338
ITS1-107R	CTGACCAAATGTGTTAGTTGTGAG	ITS1

Each PCR run included a sample containing DNase-free water as a negative template control. The risk of sample cross-contamination was minimized by following standard PCR procedures. All PCR products were purified by use of the DNA Clean and Concentrator-5 Kit (Zymo Research, Orange, CA, USA) and sequenced directly. In direct sequencing of samples containing two or more trinucleotide repeat populations, the minority populations are three bases out of alignment with the majority population, which can be directly observed on the sequence chromatograms (Figure 2). The sequences flanking the repeat region were determined accurately by sequencing from two directions. To further confirm the presence of heterogeneous repeat populations, some PCR products were sequenced after subcloning by use of the pPCR-Script Amp Cloning Kit (Stratagene, La Jolla, CA, USA). Recombinant plasmids were transformed into SURE competent cells (Stratagene), which permit cloning of unstable DNA. For specimens containing mixed STR alleles, the proportion of individual alleles were arbitrarily estimated by the height of the sequence chromatogram peaks and/or by the cloning results if available.

### rRNA-SNP typing

We previously identified 6 *M. genitalium *genotypes based on sequence analysis of two fragments of the rRNA operon, including a fragment of approximately 800 bp covering parts of the 16S and 23S rRNA genes and the internal transcribed spacer between them (16S-ITS1-23S) and a fragment of approximately 450 bp covering parts of the 23S and 5S rRNA genes and the ITS between them (23S-ITS2-5S) [13]. Types 1–5 were identified by four SNPs located within the 16S-ITS1-23S fragment. Type 6 was differentiated from Type 2 by a single SNP at position 124 in the ITS2 region and was present in only 2/31 patients. We amplified and sequenced the 23S-ITS2-5S region in 10 Scandinavian patients with the type 2 genotype and none of them had the SNP characteristic of Type 6. Therefore, given the low discriminatory value of Type 6, in this study we sequenced only the 16S-ITS1-23S fragment in order to identify Types 1–5. Details of the PCR reaction and sequencing methods have been published [13].

Because we found that rRNA-SNPs typing may be useful for the study of the geographic distribution of *M. genitalium*, we developed a SSCP method to permit rapid detection of the 3 most common types. The SSCP method was developed using the specimens from the 31 unrelated New Orleans patient isolates for which the 16S-ITS1-23S sequences had been determined previously as noted above [13]. SSCP conditions were optimized using the GeneGel SSCP Starter Kit according to the instructions of the manufacturer (Amersham Pharmacia Biotech, San Francisco, CA, USA). We performed nested PCR for all specimens, using primers Mg16-1240F and Mg23-2033R for the first round of amplification and primers Mg16-1381F [13] and ITS1-107R for the second round (Table 3). Four-microliter aliquots of the nested-PCR products (246 bp) were mixed with 4 μl of loading buffer containing 90% formamide, 0.025% (wt/vol) bromophenol blue, 0.025% (wt/vol) xylene cyanol, and 3% (vol/vol) glycerol. This mixture was heated at 95°C for 5 min and then chilled in an ice water bath. Five-microliter mixture was loaded on a precast GeneGel SSCP gel (Amersham Pharmacia Biotech). Electrophoresis was performed using the temperature-controlled Gene-Phor Electrophoresis System and SSCP running buffer C, pH 8.3 (Amersham Pharmacia Biotech). The optimal conditions included a running temperature of 12°C, and a running voltage at 80 V for 20 min followed by 510 V for 140 min. The gels were stained by using the PlusOne DNA Silver Staining Kit (Amersham Pharmacia Biotech).

### MG191-SNP typing

PCR amplification and sequencing of the MG191 conserved region AB (approximately 280 bp) were performed as described previously [10].

### Bioinformatics tools

Sequence analysis was done using the Chromas 1.45 (Technelysium Pty Ltd, Tewantin, Australia) and CLC Combined Workbench 1.0.2 (CLC bio, Aarhus, Denmark). Discriminatory index was calculated according to the method described previously [35]. To evaluate the relatedness of M. genitalium strains, clustering analysis was performed by use of the BioNumerics 5.0 software (Applied Maths, Sint-Martens-Latem, Belgium), in which heterozygous genotypes and the frequency of each genotype are taken into account. Dendrograms were constructed by the unweighted pair-group method using arithmetic averages (UPGMA) method. All statistic tests were carried out using SAS 9.1 (SAS Institute Inc., Cary, NC). The distribution of M. genitalium genotypes between New Orleans and Scandinavia was analyzed by Fisher's exact test. The correlation between the repeat copy number and prevalence of mixed alleles of PLP STRs was assessed by Spearman's correlation test.

### Nucleotide sequence accession numbers

Representative nucleotide sequences obtained in this study have been deposited in the GenBank under accession numbers DQ470670 and DQ470674 for MG307-STRs, DQ470672 and DQ470673 for MG338-STRs, and DQ470671 and DQ470675 for MG185 sequences, and EU131379 to EU131382 for the sequence types 57 to 60 of the MG191 conserved AB region.

## Authors' contributions

LM contributed to experimental design, bioinformatics analysis, and manuscript writing. SNT and RL supervised the collection of the New Orleans patient specimens and clinical data and participated in data analysis. JSJ provided Scandinavian patient specimens and data about most of the MG191-SNP typing, and was involved in data analysis and manuscript editing. LM (Tulane) performed the statistical analysis. DHM participated in coordination and helped to edit the manuscript. All authors read and approved the final manuscript.

## Supplementary Material

Additional file 1 Relationship between repeat copy number and prevalence of mixed STR alleles. Click here for file

Additional file 2 Analysis of plasmid clones from selected *M. genitalium *specimens. Click here for file

Additional file 3 Discriminatory power of various genetic markers for *M. genitalium*. Click here for file
