# Supplementary material for: Short tandem repeat sequences in the Mycoplasma genitalium genome and their use in a multilocus genotyping system
Source: BMC Microbiol. 2008 Jul 29;8:130. doi: 10.1186/1471-2180-8-130 (PMC2515158; doi:10.1186/1471-2180-8-130)
Supplement: Additional file 1 — Relationship between repeat copy number and prevalence of mixed STR alleles. [file 1471-2180-8-130-S1.doc]

**Additional file 1**

MG307

MG338

MG309

**Relationship between repeat copy number and prevalence of mixed STR alleles at three lipoprotein genes MG307, MG309 and MG338**. The Spearman’s correlation coefficient is 0.95 (p < 0.0001) for MG307, 0.26 (p > 0.45) for MG309, and 0.87 (p < 0.0001) for MG338. The Spearman’s partial correlation coefficient (controlling for total number of specimens) is 0.95 (p < 0.0001) for MG307, 0.26 (p > 0.45) for MG309, and 0.88 (p < 0.0001) for MG338.
