# Supplementary material for: Short tandem repeat sequences in the Mycoplasma genitalium genome and their use in a multilocus genotyping system
Source: BMC Microbiol. 2008 Jul 29;8:130. doi: 10.1186/1471-2180-8-130 (PMC2515158; doi:10.1186/1471-2180-8-130)
Supplement: Additional file 2 — Analysis of plasmid clones from selected M. genitalium specimens. [file 1471-2180-8-130-S2.doc]

**Additional file 2**

### Analysis of plasmid clones from selected *M. genitalium* specimens.

| Specimen^a^ | Gene | No. of repeats by direct sequencing | No. of clones analyzed | No. of clones (for each repeats) | | | |
| --- | --- | --- | --- | --- | --- | --- | --- |
| 7NO | MG338 | 14, 13 | 4 | 3 (14) | 1 (13) |  |  |
| 19NO | MG309 | 11, 10 | 4 | 3 (11) | 1 (10) |  |  |
| 19NO | MG338 | 6, 8 | 10 | 7 (6) | 1 (8) | 1 (7) | 1 (5) |
| 57 | MG309 | 12, 11 | 8 | 5 (12) | 3 (11) |  |  |
| 64.1 | MG309 | 12, 11 | 9 | 7 (12) | 1 (11) | 1 (10) |  |
| 64.2 | MG309 | 12, 11 | 16 | 10 (12) | 6 (11) | 1 (10) |  |
| 120 | MG307 | 10, 9, 8 | 6 | 3 (10) | 2 (9) | 2 (8) | 2 (11) |
| 123.1 | MG307 | 5 | 12 | 12 (5) |  |  |  |
| 123.2 | MG307 | 5, 6 | 11 | 10 (5) | 1 (6) |  |  |
| 129.1 | MG309 | 16, 15 | 6 | 4 (16) | 2 (15) |  |  |
| 129.2 | MG309 | 16, 15 | 7 | 6 (16) | 1 (15) |  |  |
| 154 | MG338 | 9, 8 | 4 | 3 (9) | 1 (8) |  |  |
| 198.1 | MG309 | 15, 16, 17 | 19 | 14 (15) | 3 (16) | 2 (17) |  |
| 198.2 | MG309 | 15, 16, 17 | 4 | 2 (15) | 1 (16) | 1 (17) |  |

^a^ The number following the decimal point indicates patient visit number with 1

representing the first visit and 2 the second visit. For specimens containing mixed

sequences, the genotypes are listed in the order from predominant to minor type.
