# Supplementary material for: Short tandem repeat sequences in the Mycoplasma genitalium genome and their use in a multilocus genotyping system
Source: BMC Microbiol. 2008 Jul 29;8:130. doi: 10.1186/1471-2180-8-130 (PMC2515158; doi:10.1186/1471-2180-8-130)
Supplement: Additional file 3 — Discriminatory power of various genetic markers for M. genitalium. [file 1471-2180-8-130-S3.doc]

**Additional file 3**

**Discriminatory power of various genetic markers for *M. genitalium.***

| Genetic marker | Polymorphism type | No. of genotypes^a^ | Diversity index |
| --- | --- | --- | --- |
| **Single marker** |  |  |  |
| MG307 | STR | 4 | 0.7381 |
| MG309 | STR | 10 (18^b^) | 0.9153 (0.9471^b^) |
| MG338 | STR | 8 | 0.8730 |
| rRNA | SNP | 3 | 0.5820 |
| MG191 | SNP | 16 | 0.9392 |
| **Selected combinations of two markers** | | | |
| MG307 + MG309 | STR | 20 | 0.9788 |
| MG307 + MG338 | STR | 19 | 0.9656 |
| MG309 + MG338 | STR | 21 | 0.9762 |
| MG309 + rRNA | STR + SNP | 19 | 0.9683 |
| MG191 + rRNA | SNP | 20 | 0.9709 |
| MG309 + MG191 | STR + SNP | 20 (26 ^b^) | 0.9894 (0.9947^b^) |

^a^Based on data from 28 unrelated specimens containing a single allele in all 5 loci tested.

^b^When both the repeat number and AGT/AAT distribution pattern variations at MG309 were taken in account.
